# Adherence to coprescribing of laxatives with opioids and associated characteristics in general practices in the Netherlands

**DOI:** 10.1186/s12875-022-01911-8

**Published:** 2022-12-05

**Authors:** Karin Hek, Fouzia Lghoul-Oulad Saïd, Joke C. Korevaar, Linda E. Flinterman, Liset van Dijk, Patricia M. L. A. van den Bemt

**Affiliations:** 1 Nivel, Netherlands Institute for Health Services Research, PO box 1568, 3500 BN Utrecht, The Netherlands; 2 Division of BioTherapeutics, Leiden Academic Centre for Drug Research (LACDR), Gorlaeus Laboratories, Leiden University, Leiden, The Netherlands; 3 Department of Clinical Pharmacy and Pharmacology, University Medical Center Groningen, Groningen, The Netherlands; 4 Department of Hospital Pharmacy, Erasmus University Medical Center, Rotterdam, The Netherlands; 5 Department of PharmacoTherapy, -Epidemiology and -Economics (PTEE), Groningen Research Institute of Pharmacy, Faculty of Science and Engineering, University of Groningen, Groningen, the Netherlands

**Keywords:** Adverse drug event, Analgesics, Guideline adherence, Laxatives, Opioids, Primary care

## Abstract

**Background:**

Guidelines recommend to prescribe a laxative with an opioid to prevent constipation. We aimed to determine the adherence by general practitioners (GPs) to this recommendation and to explore which GP- and patient related factors were associated with it from the perspective of the GP.

**Methods:**

We conducted an observational study using GPs’ prescription data from the Nivel Primary Care Database combined with a questionnaire asking for reasons of non-adherence. The proportion of first opioid prescriptions prescribed together with a laxative was determined as primary outcome. Possible explanatory factors such as the quality of registration, the level of collaboration with the pharmacy, familiarity with the recommendation and use of a clinical decision support system were explored, as were the self-reported reasons for non-adherence (classified as either GP-related or patient-related). We assessed the association of factors with the primary outcome using univariable multilevel logistic regression analysis.

**Results:**

The recommendation was measured in 195 general practices. The median proportion of first opioid prescriptions prescribed together with a laxative in these practices was 54% (practice range 18–88%). None of the determinants was consistently associated with the primary outcome. GPs from 211 practices filled out the questionnaire and the most frequently mentioned reason not to prescribe a laxative was that the patient has laxatives in stock, followed by that the patient doesn’t want a laxative; both were patient-related factors.

**Conclusion:**

There was room for improvement in following the guideline on laxative prescribing in opioid use. A main reason seemed to be that the patient refuses a laxative. Improvement measures should therefore focus on communication between GPs and patients on the relevance of co-using a laxative with opioids. Future studies need to establish the effect of such improvement measures, and determine whether reasons for non-adherence to the guideline changed over time.

## Background

Opioids offer pain relief for severe acute and moderate to severe chronic pain [1]. Alongside this pain relief, up to 80% of opioid users also experience at least one adverse event [2]. The most common adverse event is opioid-induced-constipation (OIC) [2, 3]. Studies have reported frequencies of OIC varying from 15 to 95% [2, 4–9]. This wide range can be attributed to many factors including differences in study protocol, patient population and included opioids. Constipation has potentially serious complications such as haemorrhoid formation, rectal pain, bowel obstruction and even death [10]. Moreover, OIC causes significant distress, reduces work productivity, lowers health-related quality of life and increases opioid non-adherence [11].

To prevent OIC, guidelines recommend to prescribe laxatives in all patients starting with an opioid, unless contra-indicated (e.g. in case of acute abdominal pain of unknown cause, intestinal obstruction or diarrhoea) [12]. Non-adherence to these guidelines may lead to unnecessary hospital admissions. In a recent Dutch study, opioid-induced constipation was responsible for 7.5% of all preventable hospital admissions related to medication [13]. Non-adherence to guidelines on laxative prophylaxis in opioid use has been described in literature. A Dutch study among patients in a community pharmacy setting showed that in 2002 only 37% of patients who received an opioid also started taking laxatives within 5 days [14], whilst in 2012 another Dutch study showed that 54% of opioid users used a laxative [15]. In a more recent study among opioid initiators in the Netherlands, concomitant use of laxatives was found to be 74.8% [16]. Similar percentages have been reported in studies performed in the United States (55% [17] and 23% in patients with lung cancer [18]) and Switzerland (50% in noncancer and 67% in cancer populations) [19] Non-adherence to coprescribing of laxatives in opioid therapy remains an issue up to this day, as a recent study in palliative care has shown: one third of patients receiving opioids in the last year of their lives were not prescribed laxatives [20].

The main reason not to use laxatives in the recent Dutch study was that patients did not consider them necessary [16], but the underlying motivation for these perceptions remains unclear as well as the perceptions of prescribers. Non-adherence to guidelines by physicians may be caused by factors such as lack of agreement with guideline recommendations [21, 22], lack of outcome expectancy or self-efficacy [23], external limitations (e.g. time, tools) [23] and/or recommendations not applicable to specific patients [21, 22]. Whether these reasons also apply to non-adherence to guidelines on laxative prescribing in opioid users is unclear. A recent Italian study used a survey to explore the practice of opioid and laxative prescribing, but underlying motivation was not studied, neither in the original study [24] nor in the follow-up study [25].

Since most opioids are prescribed by general practitioners (GPs) insight into their perceptions on guideline based co-prescribing of laxatives in opioid users is especially important. Therefore, the aim of this study was to determine how often laxatives were co-prescribed with opioids in general practice, what factors (both GP-related and patient-related) were associated with adherence and what the reasons were that GPs mentioned for non-adherence to co-prescribing of laxatives.

## Methods

### Study design

This was an observational study using prescription data from general practitioners and a questionnaire among general practitioners.

### Data source

This study was performed using data from the Nivel Primary Care Database (Nivel-PCD). Nivel-PCD is a dynamic longitudinal database that collects routinely registered data from electronic health records (EHRs) of approximately 500 general practices. Data include information on patient characteristics, consultations, morbidity, and prescriptions. A research data set can be requested under conditions. According to Dutch legislation, neither obtaining informed consent from patients nor approval of a medical ethics committee is obligatory for this type of observational studies containing no directly identifiable data (Dutch Civil Law, Article 7:458). This study was approved by the applicable governance bodies of Nivel Primary Care Database under number NZR00315.024. For this study we used data from:

1. questionnaires sent to 444 practices participating in Nivel-PCD and filled out by 211 practices and
2. EHR-data of the years 2013 and 2014 from a subsample of 195 general practices participating in Nivel-PCD who fulfilled quality criteria on completeness of prescription data in both years (i.e. at least 46 weeks of prescription data per practice).

Both questionnaire-data and EHR-data were available from a subsample of 103 practices.

### Guideline adherence

The Dutch primary care guideline on constipation [26] nowadays states that a laxative should be co-prescribed when starting with an opioid; the guideline stated this at the time of the study as well [27].

Guideline adherence per practice was measured using prescription data from EHRs. To determine adherence, we defined the following indicator for the proportion of first opioid prescriptions together with a laxative, which was derived from a previous study [13]:

- Denominator: Number of patients with a first prescription of an opioid in 2014.
- Numerator: Number of patients that received a laxative together with the first opioid prescription.

Per general practice the indicator was calculated as numerator/denominator *100. The first prescription was defined as the first opioid prescription in 2014 with no opioid prescription in the six months before. To check this we also used prescription data from 2013. Treatment with a laxative from the start of an opioid was determined as a laxative prescription in the 90 days before or 15 days after the first opioid prescription. Since patients could use laxatives intermittently this range was used to account for prior prescription of laxatives that had not been used or are still used and prescription of laxatives during the period after the start of the opioid, which is often agreed upon with the patient.

Prescriptions were coded using the Anatomical Therapeutical Chemical (ATC) classification system [28]. Opioids were defined as ATC-codes N02AA (excluding N02AA55, N02AA59 and N02AA79), N02AB, N02AC, N02AD, N02AG, N02AE or N07BC01. Laxatives were defined as ATC-codes A06A, A02AA02, A02AA03, or A02AA04. Patients who used antipropulsives (ATC-code A07DA) in 2014 were excluded, as diarrhea is a reason not to prescribe a laxative.

### Questionnaire to general practitioners (GPs)

To determine the reasons for not co-prescribing a laxative with opioids, we sent an online questionnaire to 444 general practices participating in Nivel–PCD in December 2015. We asked questions on the following topics:

- Use of a clinical decision support system which provides the GP with a recommendation to prescribe a laxative when prescribing an opioid;
- Participation in pharmacotherapy audit meetings (PTAMs; i.e. local peer review groups existing of GPs and community pharmacists who regularly meet to discuss pharmacotherapy issues and quality of prescsribing [29]);
- Collaboration with the pharmacy;
- The GP’s familiarity and acceptance of the guideline recommendations;
- Three most common reasons for non-adherence to the recommendations; these reasons could be related to GP-factors (e.g. not convinced of medical necessity) or patient-factors (e.g. not willing to take laxatives). We prespecified 13 possible reasons for non-adherence, and GPs could add reasons in an open text field. The reasons to choose from were: patient still has laxatives, patient does not want laxatives, patient already uses a lot of medication, allergic to laxatives, contra-indications for laxative use, patient cannot take laxatives (e.g. in case of palliative sedation), I prescribe laxatives later during opioid treatment if necessary, in case of short-term or on demand opioid use, in case of very low opioid dosages, patient will use over-the-counter medication to prevent constipation, in case of acute administration of an opioid (e.g. for transportation to an emergency room), no medical necessity, or other, to be filled in by the GP. The reasons were classified into GP-related and patient-related after analysis of the questionnaire results.

The questionnaire was to be filled out by one GP per practice. A paper-questionnaire was sent in case of non-response after four weeks.

### Explanatory factors

*Quality of registration*: we determined the quality of registration using two indicators from a previously developed EHR-scan[30], that was specifically developed to assess the quality of registration in EHRs: 1) the percentage of episodes with a meaningful diagnosis code, which indicates whether a GP adds a relevant diagnosis code to an episode and 2) the percentage of contacts that were linked to an episode, which provides information on the completeness of diagnosis registration.

*Collaboration with the pharmacies*: in the questionnaire we asked whether GPs have agreements with pharmacies on: I) medication monitoring (regarding amendments in drug prescriptions; registration of morbidities; determination of or doubt on patient adherence; initiation of automated dose dispensing; and conducting a medication review), II) patient counseling (who gives what information on medication), and III) collaboration (whether there are agreements on: medication of preference for specific indications; information exchange of patient’s diagnosis; laboratory measurements, and procedure on medication transfer when patient is moved from/to a healthcare facility). When the GP had an in-house pharmacy this was registered as well. In addition, we included information on participation in PTAMs. We also asked whether co-prescription of a laxative with an opioid was discussed during these meetings (yes/no) and when (in or before 2014).

*Familiarity with and acceptance of the guideline recommendations*: from the questionnaire we extracted whether the GP knows the guideline (yes/no) and whether he or she thinks it is useful to always co-prescribe a laxative with an opioid (yes/no). If not, we asked for the reasons why not.

*Use of a clinical decision support system*: GPs indicated how often they use a clinical decision support system when prescribing medication (never to hardly ever/in less than half of the prescriptions/in half or more than half of the prescriptions/always).

### Outcome measures

The primary outcome of this study was the proportion of first opioid prescriptions prescribed together with a laxative. We also assessed determinants associated with adherence to the guideline recommendations, and reasons the GPs gave for non-adherence to the guideline recommendation.

### Statistical analysis

The primary outcome was calculated for each practice. We performed univariable multilevel logistic regression analysis to determine the association between the potentially explanatory factors described above and the proportion of first opioid prescriptions prescribed together with a laxative, accounting for the nesting of patients (level 1) within practices (level 2). We applied a Bonferroni correction to adjust for multiple testing. Analyses were performed using Stata version 14.0. The results were presented as odds ratios (OR) and 95% confidence intervals (95% CI). Furthermore the reasons to deviate from the guideline were stratified by high and low adherence.

## Results

### Adherence per practice

**Fig.1:**
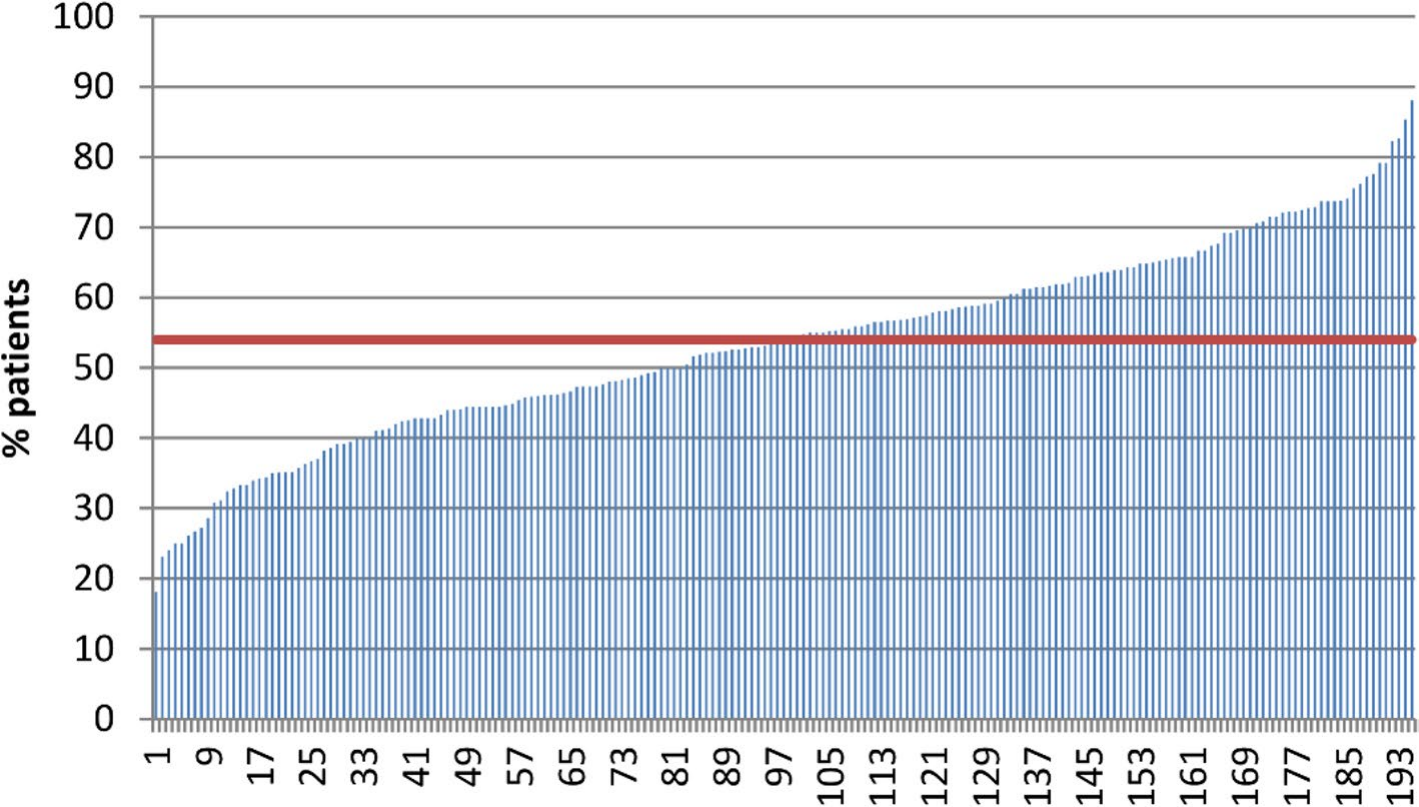
Percentage of patients with a first opioid prescription and a laxative coprescribed by practice; Horizontal line = median of all practices

The number of patients per practice with a first opioid prescription in 2014 ranged from 6 to 220, with a median of 38 patients per practice. A median of 20 patients per practice was co-prescribed a laxative with their opioid (range 3–142 patients). The recommendation to co-prescribe a laxative was followed in 18% to 88% patients per practice, with a median of 54% patients (5–95% range: 31–76%; Fig. 1).

### Potentially explanatory factors for non-adherence and their association with adherence

In total, 211 GPs (one per practice) filled out the questionnaire (response rate 48%). EHR data were available from 103 of these practices in 2013 and 2014. The GPs who filled out the questionnaire did not significantly differ in adherence to the guideline from GPs who did not fill out the questionnaire (median percentage of guideline adherence was 54% in both groups).

### Explanatory factors associated with adherence

Almost all GPs (99%) were familiar with the recommendation to co-prescribe a laxative with an opioid and 89% also agree with this recommendation (Table 1). Regarding the quality of registration, half of the practices provided a meaningful diagnosis to 97% of episodes and linked 97% of the contacts to an episode. In addition, almost all GPs participated in PTAMs. However, the number of procedures performed in collaboration with pharmacies on topics such as medication monitoring, and patient counseling differed considerably across general practices. Eighteen percent of the practices collaborated with the pharmacy on half or less of the topics covered in the questionnaire. Use of a clinical decision support system also varied between GPs. While one-fifth of the GPs always used a clinical decision support system, one-third hardly ever used such a system.

**Table 1:** Practice scores in factors potentially explaining nonadherence to the recommendation to prescribe a laxative with an opioid

Potentially explanatory factors	
Quality of registration	
% of episodes with a meaningful diagnosis, median (10–90%)	96.9 (69.8–99.9)
% of contacts linked to an episode, median (10–90%)	96.5 (78.3–99.3)
Collaboration	
Participation in pharmacotherapy audit meetings (%)	
Yes	97.1
No	2.9
Number of procedures with pharmacist (%)	
0–5 topics	18.4
6–8 topics	45.6
9–10 topics	35.9
Agreement with pharmacist on coprescribing laxative (%)	
Yes	80.6
No	16.0
GP with an in-house pharmacy	3.4
Use of an electronic clinical decision support system (%)	
Always	21.1
In half or more than half of all prescriptions	18.7
In less than half of all prescriptions	27.3
Never to hardly ever	33.0
Familiar with guideline recommendations (%)	
Yes	99.5
No	0.5
Agree with guideline recommendations (%)	
Yes	88.9
No	11.1

Two of the explanatory factors (*Episodes with a meaningful diagnosis* and *Contacts linked to an episode)* were related with adherence to the recommendation to prescribe a laxative when prescribing an opioid after bonferroni correction (see Table 2). Both significantly associated explanatory factors are indicators for the quality of registration, but showed contradictory results with higher adherence when more *episodes with a meaningful diagnosis* were registered (odds ratio of 1.59 (1.25–2.01)) and lower adherence when more *contacts were linked to an episode* (odds ratio of 0.61 (0.47–0.80))*.*

**Table 2:** Association between potentially explanatory factors and the proportion of first opioid prescriptions prescribed together with a laxative^a^

	OR (95% CI)	B(SE)	*P*-value	McKelvey & Zavoina Pseudo *R*^2^
Quality of registration				
Episodes with a meaningful diagnosis				0.047
0–70% of episodes	ref			
More than 70% of episodes	1.59 (1.26–2.01)	0.46 (0.12)	** < 0.001**	
Contacts linked to an episode				0.047
0–80% of contacts	ref			
More than 80% of contacts	0.61 (0.47–0.80)	-0.49 (0.14)	** < 0.001**	
Collaboration				
Participation in pharmacotherapy audit meetings				0.043
Yes	ref			
No	1.20 (0.68–2.10)	0.18 (0.29)	0.53	
Number of procedures with pharmacist				0.043
0–5 topics	ref			
6–8 topics	1.05 (0.77–1.41)	0.046 (0.15)0.13 (0.16)	0.77	
9–10 topics	1.13 (0.83–1.55)		0.43	
Agreement with pharmacist on coprescribing laxative				0.045
No	ref			
Yes	1.32 (0.92–1.89)	0.28 (0.18)	0.13	
Use of an electronic clinical decision support system (%)				
Always	ref			0.044
In half or more than half of all prescriptions	0.98 (0.70–1.38)	-0.019(0.17)-0.11 (0.16)-0.23	0.91	
In less than half of all prescriptions	0.89 (0.65–1.22)	(0.16)	0.49	
Never to hardly ever	0.80 (0.58–1.09)		0.16	
Agree with guideline recommendations				
Yes	ref			0.044
No	1.56 (0.93–2.62)	0.44 (0.26)	0.091	

In bold: significantly associated based on bonferroni corrected *p*-value of 0.0071

^a^Each of the variables was tested in separate multilevel analyses, familiarity with the recommendation was not tested, as almost all GPs were familiar with the recommendation

### Reasons for non-adherence to the recommendation

The most frequently mentioned reason not to prescribe a laxative was that the patient still has laxatives in stock, followed by that the patient does not want a laxative and that the patient has a contraindication (Table 3). These were all patient-related factors. We compared reasons not to co-prescribe a laxative between GPs who often co-prescribed a laxative (highest quartile) and GPs who did this relatively less (lowest quartile). Compared to GPs in the highest quartile, GPs in the lowest quartile more often mentioned not to co-prescribe a laxative when the patient uses a very low opioid dose as top 3 reason not to co-prescribe (20.7% versus 4% in the highest quartile). GPs in the highest quartile of co-prescribing a laxative more often mentioned as a top 3 reason not to co-prescribe: intermitted use of opioids (32% versus 24.1% in the lowest quartile), that they will prescribe a laxative later (24% versus 13.8% in the lowest quartile) and that the patient cannot use a laxative e.g. because the patient is in the palliative phase (40% versus 31% in the lowest quartile). Not prescribing a laxative because of low or intermittent dosing of the opioid was classified as a GP related factor, because this is a personal interpretation of the guideline (which states that laxatives should always be started). A patient not being able to take a laxative was classified as a patient-related factor.

**Table 3:** Most common reasons for 211 GPs not to co-prescribe a laxative with an opioid in total and for GPs scoring in the highest and lowest quartile of adherence

	**% of GPs with reason in top-3**		
**Reason not to adhere to recommendation**	**All GPs (*n* = 211)**	**Score in lowest quartile of guideline adherence (*n* = 29)**^a^	**Score in highest quartile of guideline adherence (*n* = 25)**^a^
Patient still has laxative	58.8	69.0	72.0
Patient does not want laxative	44.6	34.5	36.0
Contra-indication of laxative use	39.8	51.7	48.0
Patient cannot take laxative (e.g. palliative phase)	33.7	31.0	40.0
Intermitted opioid use (when needed)	28.0	24.1	32.0
Acute opioid use (e.g. transportation to ER)	22.3	24.1	20.0
GP will prescribe laxative later, if necessary	17.5	13.8	24.0
Patient will use over the counter laxative to prevent constipation	12.3	6.9	4.0
Very low dose of opioid	9.5	20.7	4.0
Adverse events of laxative	7.6	3.5	8.0

^a^From a selection of 103 practices with both guideline adherence scores and information from the questionnaire

## Discussion

In Dutch primary care, laxatives are co-prescribed with the first prescription of an opioid in 18 to 88% of patients per general practice with a median of 54%. This broad practice range suggests that there is ample room for improvement of adherence to this recommendation. We tested several possible factors that may be associated with adherence to the recommendation. None of the studied factors was consistently associated with adherence. Patient specific reasons seem more important than general factors such as collaboration with the pharmacy.

The level of adherence in this study is in line with or slightly higher than in older studies both in the Netherlands [14, 15] and outside [17–19], while more recent studies tend to show higher levels of adherence [16, 20] However, while these studies only looked at overall adherence, we investigated practice variation. Since the upper level of the range of adherence is 88%, an increase in the overall level of adherence seems achievable. The level of adherence in this study took into account two valid reasons not to prescribe a laxative, namely that the patient still had a laxative in stock and that the patient had a contra-indication (i.e. diarrhea). Therefore, these reasons, do not explain the low percentage of adherence found in this study.

Overall, the quality of registration was high. The assumption was that if the quality weakens it could have negative consequences on the adherence, because emergency GPs could base their healthcare decisions on incomplete patient records. This could lead to incorrect assumptions and therefore incorrect care for the patients. The high registration quality may explain the contradictory results on the two indicators, as the number of cases within the low quality group is very low and thus the results are not robust. Given these contradictory results and the already high quality of registration in general, this factor does not need to be the focus of specific improvement measures.

Although the recommendation to co-prescribe a laxative with an opioid is known and accepted by almost all GPs, they do not always adhere to it. One of the most common reasons for non-adherence provided by GPs is that the patient does not want the laxative. However, not using a laxative with opioids carries a risk for the patient, of which patients should be well informed. Especially since OIC carries a risk of hospitalisation [31]. The study by De Bruin et al [16] confirmed that patients are not always convinced they need laxatives when using opioids, further stressing the need for patient information.

A survey performed in noncancer patients with chronic pain showed that 79.8% of the interviewed patients were not comfortable discussing their constipation and only 20% would talk to their physician about this topic [32]. Patients may not be aware that their constipation was caused by the opioid and do not bring this up during a consultation due to embarrassment. Patients may also experience paradoxical diarrhea (overload diarrhea) and not recognize their constipation. Patients should therefore be frequently asked and updated on OIC to prevent unnecessary distress and harm.

Another reason mentioned was that the patient is not capable of swallowing a laxative. This is most often the case in palliative patients who can especially benefit from a laxative [33]. Furthermore, GPs mentioned they start with a low dose of an opioid assuming the risk of constipation declines with reduction of the dose. However, constipation may occur at opioid doses lower than those required for analgesia. Thus, merely lowering the opioid dose may not be effective for managing OIC, while the analgesic benefit of the prescribed opioid may be reduced or lost [34]. Further educating the GPs on this point could improve patient’s health.

Recommendations always leave room for shared decision making with the patient. We did not study how often the choice not to prescribe a laxative was a result from well-informed and shared-decision making.

### Strengths and limitations

The main strength of the study is the broad range of general practices included in this study, as well as the primary care setting. The number of studies on laxative co-prescribing in opioid users is rather limited, so this study adds important information to the existing evidence base and practice variation.

A few limitations need to be discussed as well. Both the registration data and the data from the questionnaire were analyzed on practice level. However, questionnaires were answered by one GP per practice, whereas 88% of the practices consist of two or more GPs. The GP’s answer may not always reflect the opinion and experience of all GPs in the practice. In the current study it was not possible to assess adherence on GP level.

The data used for this study are not recent (2014), but do show an overview of the adherence at that time. Looking at more recent literature, it can be assumed that adherence has improved since then, but that non-adherence is still prevalent [16, 20]. We think it is unlikely that the reasons for non-adherence changed much over the past few years, as the context of prescribing, such as guidelines, has not changed, no national campaigns have been launched on this topic and behavioral change is typically a slow process. Furthermore, we found that patient specific factors seem more important reasons for non-adherence, than practice related factors, such as quality of registration or collaboration with the pharmacy, that are more likely to have changed over time. So the results of our study can still be used to optimize guideline adherence. We do recommend future studies, however, in order to establish whether reasons indeed remain the same, but especially to determine the effect of interventions focused on the reasons for non-adherence. It is possible that a selection of more adherent GPs filled out the questionnaire. However, there was no difference between GPs who did and who did not respond to the questionnaire in adherence to the studied recommendation. The influence of selection bias thus appears to be limited.

Our focus in this study was to determine the reasons GP deviate from the guideline on co-prescribing laxatives with opioids. They mentioned rejection by patient as one of the reasons, but we did not explore why the patient would reject treatment. Another reason we did not explore was whether the contra-indications mentioned were justified to reject a laxative nor did we determine whether GPs were more adherent with patients at higher risk of OIC, such as females or age ≥ 50 years [35]. Further research is needed to determine this.

Finally, the reasons for non-adherence were responses to a relatively general questionnaire and thus lack important details such as why patients would still have laxatives in stock. Future studies using in-depth interviews with both patients and GPs need to explore the reasons in more detail.

## Conclusion

Given the level of adherence to the recommendation to combine an opioid with a laxative and the large practice variation, there is ample room for improvement. GPs are aware of the recommendation to combine a laxative when initiating an opioid, and mentioned the patient not wanting a laxative as an important reason. Improvement measures should therefore focus on better communication with patients on the relevance of co-using a laxative when using opioids.

## Data Availability

The data underlying this article will be shared under conditions applicable to Nivel-PCD data and on reasonable request to the corresponding author.

## Abbreviations

ATC: Anatomical Therapeutic Chemical classification system
EHR: Electronic Health Record
GP: General practitioner
Nivel-PCD: Nivel Primary Care Database
OIC: Opioid-induced constipation
OR: Odds ratio
PTAM: Pharmacotherapeutic audit meetings
